# *Glomus mosseae* improved the adaptability of alfalfa (*Medicago sativa* L.) to the coexistence of cadmium-polluted soils and elevated air temperature

**DOI:** 10.3389/fpls.2023.1064732

**Published:** 2023-03-09

**Authors:** Yun-feng Gao, Xia Jia, Yong-hua Zhao, Xiao-yi Ding, Chun-yan Zhang, Xiao-juan Feng

**Affiliations:** ^1^ Shaanxi Key Laboratory of Land Consolidation, School of Land Engineering, Chang’an University, Xi’an, China; ^2^ Key Laboratory of Subsurface Hydrology and Ecological Effects in Arid Region of Ministry of Education, School of Water and Environment, Chang’an University, Xi’an, China

**Keywords:** antioxidants, antioxidant enzymes activities, chelators, osmotyes, gene expression

## Abstract

The coexistence of heavy metal-polluted soils and global warming poses serious threats to plants. Many studies indicate that arbuscular mycorrhizal fungi (AMF) can enhance the resistance of plants to adverse environments such as heavy metals and high temperature. However, few studies are carried out to explore the regulation of AMF on the adaptability of plants to the coexistence of heavy metals and elevated temperature (ET). Here, we investigated the regulation of *Glomus mosseae* on the adaptability of alfalfa (*Medicago sativa* L.) to the coexistence of cadmium (Cd)-polluted soils and ET. *G. mosseae* significantly enhanced total chlorophyll and carbon (C) content in the shoots by 15.6% and 3.0%, respectively, and Cd, nitrogen (N), and phosphorus (P) uptake by the roots by 63.3%, 28.9%, and 85.2%, respectively, under Cd + ET. *G. mosseae* significantly increased ascorbate peroxidase activity, peroxidase (POD) gene expression, and soluble proteins content in the shoots by 13.4%, 130.3%, and 33.8%, respectively, and significantly decreased ascorbic acid (AsA), phytochelatins (PCs), and malondialdehyde (MDA) contents by 7.4%, 23.2%, and 6.5%, respectively, under ET + Cd. Additionally, *G. mosseae* colonization led to significant increases in POD (13.0%) and catalase (46.5%) activities, *Cu/Zn*-superoxide dismutase gene expression (33.5%), and MDA (6.6%), glutathione (22.2%), AsA (10.3%), cysteine (101.0%), PCs (13.8%), soluble sugars (17.5%), and proteins (43.4%) contents in the roots and carotenoids (23.2%) under ET + Cd. Cadmium, C, N, *G. mosseae* colonization rate, and chlorophyll significantly influenced shoots defenses and Cd, C, N, P, *G. mosseae* colonization rate, and sulfur significantly affected root defenses. In conclusion, *G. mosseae* obviously improved the defense capacity of alfalfa under ET + Cd. The results could improve our understanding of the regulation of AMF on the adaptability of plants to the coexistence of heavy metals and global warming and phytoremediation of heavy metal-polluted sites under global warming scenarios.

## Introduction

Due to having powerful tolerance to adverse environments, high accumulation of toxic elements, and large biomass (Bagavathiannan and Van Acker, 2009; Shi et al., 2017), alfalfa (*Medicago sativa* L.) is widely planted for phytoremediation of toxic element-polluted soils. The strong resistance of alfalfa to adverse environments, such as heavy metals and heat, depends on its remarkable defense system such as antioxidant enzymes, chelators, non-enzymatic antioxidants, and osmolytes (Dai et al., 2015; Wassie et al., 2019; Wang et al., 2021; Chen et al., 2022; Wang et al., 2022a). However, the defense systems may be unsuccessful when alfalfa is exposed to high heat or extra toxic elements such as lead and copper (Agnello et al., 2016; Zhang and Shen, 2017; Kareem et al., 2022), thus, some assistive measures can be used to promote its tolerance to toxic elements such as proper use of chelating agents (Chigbo and Batty, 2013), addition of mineral fertilizer (Kabir et al., 2016), and microbial inoculation (Ju et al., 2020).

Heavy metals in soils deriving from human activities distinctly affect plant growth, leading to a severe threat to agriculture (Li et al., 2014; Yang et al., 2018). Numerous studies indicate that cadmium (Cd) is high toxic, nonbiodegradable and widespread (Haider et al., 2021; Wang et al., 2022a), which poses a challenge for plant defense system. Low Cd level showed a stimulation on peroxidase (POD) and catalase (CAT) activities, chelators and osmolytes in alfalfa (Zhang et al., 2019; Wang et al., 2021; Wang et al., 2022a), however, POD and CAT activities in shoots decreased obviously with increasing Cd (Zhang et al., 2019). Therefore, the response of defense system in alfalfa to Cd stress might be associated with the dose and exposure duration.

Global warming has obvious impacts on plants (An et al., 2014; Zhao et al., 2016; Wassie et al., 2020). Global average temperature will rise by 2.1 ~ 3.5°C by the end of this century according to the prediction by IPCC (2021), which might affect alfalfa growth and defenses. Kareem et al. (2022) found that high temperature (> 45/26°C) obviously enhanced glutathione (GSH), ascorbic acid (AsA), soluble sugars, soluble proteins and malondialdehyde (MDA) contents, superoxide enzyme (SOD), POD, CAT and ascorbate peroxidase (APX) activities, and electrolyte leakage in alfalfa, however, CAT activity significantly decreased at 38/35°C (Wassie et al., 2020). In any case, photosynthetic capacity of alfalfa decreased distinctly under high temperature (Wassie et al., 2019), and the resistance to high temperature might be related to the extent of temperature rise and genotypes (An et al., 2014). Although studies focusing on individual effect of Cd and temperature on alfalfa are well conducted (Wassie et al., 2020; Wang et al., 2021; Kareem et al., 2022; Wang et al., 2022a), the coexisting effect of two factors has not been discussed in detail yet.

Arbuscular mycorrhizal fungi (AMF) can enhance the tolerance of plants to unfavorable environments by helping the roots absorb mineral nutrients and water and intercepting toxic substance (Latef et al., 2016; Dhalaria et al., 2020; Riaz et al., 2021). Many studies show that *Glomus mosseae* can effectively improve the resistance of alfalfa to unfriendly environments by forming a beneficial symbiont with the roots (Liang et al., 2019; Rahman et al., 2020b; Wang et al., 2022a). Furthermore, *G. mosseae* can decrease reactive oxygen species yield in alfalfa shoots exposed to Cd stress (Wang et al., 2021). Therefore, *G. m*osseae might be used to alleviate the stress of adverse environments, such as heavy metals and high temperature, on defense system of alfalfa.

According to the stress of elevated temperature (ET) and Cd on defense system and improvement of *G. mosseae* on the resistance of alfalfa in previous studies (Wassie et al., 2020; Rahman et al., 2020b; Wang et al., 2022a), we hypothesized that (1) *G. mosseae* colonization might improve the defense ability of alfalfa exposed to the coexistence of Cd and ET, and (2) this improvement might mitigate oxidative damage caused by the combined condition to alfalfa. Examination of the two hypotheses will improve our understanding of the regulation mechanism of AMF on the tolerance of plants to the coexistence of heavy metals and global warming and phytoremediation of heavy metal-polluted sites under global warming scenarios.

## Materials and methods

### Soil, alfalfa seeds and *Glomus mosseae* strain preparation

The soils for the experiment were collected from the topsoil (0–20 cm) at Tongchuan, China (35°17′ N, 108° 51′ E; 717 m a.s.l.) and filtrated by 2-mm sieve. The soil type was Loessal soil and the characteristics were following: total carbon (C, 21.3 g kg^-1^), total nitrogen (N, 1.3 g kg^-1^), total potassium (18.1 g kg^-1^), total phosphorus (P, 484.0 mg kg^-1^), Cd (1.43 mg kg^-1^), available N (86.2 mg kg^-1^), available potassium (162.0 mg kg^-1^), available P (15.3 mg kg^-1^), C/N ratio (16.8), and pH (7.8). The obtained soils were divided into two parts, one part was sprayed with sterilized CdSO_4_·8H_2_O solution and mixed thoroughly by hands, and the second was treated with equivalent amount of sterilized water. According to China’s soil environmental quality risk control standard for soil contamination of agricultural land (GB 15618-2018), the permissible limit concentration (at pH > 7.5) is Cd < 4.0 mg kg^-1^ DW. Thus, Cd levels consisted of Cd0 (no added Cd) and Cd1 (added 8.0 mg Cd kg dry weight soil^-1^), respectively. The treated soils were deposited in the dark for six months to achieve ionic equilibrium after being watered to 70% field water capacity. Alfalfa seeds came from Northwest A&F University, China. *G*. *mosseae* strain (BGC XJ02) was purchased from Beijing Academy of Agricultural and Forestry Sciences, China.

### Pot experiments and temperature treatments

The study was conducted on December 26, 2020. The sterilization test was performed after the stabilized soils were sterilized twice at 121°C for one hour. Then, 3.5 kg of sterilized soil per pot was used (30 cm long × 15 cm wide × 20 cm high). 12 g and 28 g of *G. mosseae* agents containing hypha and spore were spread in pots at 3 cm and 1 cm layer soil, respectively. Alfalfa seeds were sown in pots after being disinfected with H_2_O_2_ solution (10%, v/v). All procedures were performed under sterile conditions. All of pots were placed into sterile incubators (Percival E–36L2, USA) with a precise humidity and temperature regulation system. Temperature levels consisted of ambient temperature (AT) and ET (+ 3°C) according to IPCC (2021). Treatments consisted of the control (AT + Cd0), Cd (AT + Cd1), ET (ET + Cd0), ET + Cd (ET + Cd1) and ET + Cd + G.m (ET + Cd1 + *G. mosseae*), respectively. Three replicates were designed for each treatment. The average humidity in ambient and elevated incubators was set to ~ 75%. Due to the optimum temperature range (20 ~ 25°C) for alfalfa growth (Al-Hamdani and Todd, 1990), the day/night temperature in ambient and elevated incubators were designed 33/25°C and 36/28°C, respectively. The average light intensity was designed 550 μmol m^–2^ s^–1^ for a 12 h light cycle. Sixty seedlings in each pot were left for the experiment after emergence. Litter was thoroughly collected from the pots during the experiment for the analysis of plant biomass.

### Sampling and analysis of plant biomass, *G. mosseae* colonization rate in roots, and photosynthetic pigments in leaves

The whole plant was collected carefully and divided into shoots and roots after 120 day-growth. Shoots and roots were divided into two subsamples, respectively, and one subsample was dried at 60°C for the analysis of C, N, sulfur (S), P, Cd, and soluble sugars after their biomass were determined. The second subsample was stored at – 80°C for the determination of contents of phytochelatins (PCs), GSH, cysteine (Cys), ASA, MDA, soluble proteins, activities of SOD, POD, CAT, and APX, and relative expression of *Cu/Zn-SOD*, *POD*, *CAT*, and phytochelatins synthase (*PCS*) genes. Additionally, shoot samples were also used for the analysis of chlorophyll and carotenoid contents, and the roots were also used to analyze *G. mosseae* colonization rate.

Shoots and roots biomass was examined by the fresh weight method (Zhang et al., 2021). *G. mosseae* colonization rate was determined using the ink and vinegar method (Vierheilig et al., 1998). The 100 root segments (1 cm) for each sample were observed under a light microscope (×250) after being dyed. The colonization rate was calculated by a root segment colonization weighting method (Biermann and Linderman, 1981). Thus, *G. mosseae* colonization rate under ET + Cd were 18.3%. Chlorophyll and carotenoids contents were examined using the method described by Jia et al. (2016), and calculated according to Lichtenthaler and Wellburn (1983).

### Analysis of C, N, S, P, soluble sugars, and Cd contents in plants

Approximately 15 mg of shoot and root powder was used to determine C, N and S contents using an elemental analyzer (Vario Macrocube, Hanau, Germany), respectively. The P content was examined by molybdenum-antimony resistance colorimetry after shoots and roots powder was digested with concentrated sulfuric acid-hydrogen peroxide (Liu, 2001). 0.3 g of shoots and roots powder was extracted with 30 mL ethanol solution (70%, v/v) by refluxing at 80°C for 3 h, and soluble sugars contents in the filtrates were determined according to Yemm and Willis (1954). Cadmium content was determined by atomic absorption spectrophotometry (AAS, AA-7020, East-West Analysis, China) after shoots and root powder (0.2 g) was digested in the mixture of HNO_3_ and H_2_O_2_ (8:3, v/v) using a microwave digestion system (WX-6000, Shanghai Yiyao, China) (Zhang et al., 2021).

### Total RNA extraction and relative expression of *SOD*, *POD*, *CAT*, and *PCS* Genes in plants

0.3 g of fresh shoots and roots were used to extract total RNA according to the protocol described by plant total RNA extraction kit (Bioer, Hangzhou, China), respectively, which was reversely transcribed into cDNA according to the protocol provided by cDNA first-strand synthesis kit (Bioer, Hangzhou, China). Reverse transcription quantitative PCR (RT-qPCR) was used to examine relative expression of *Cu/Zn-SOD*, *POD*, *CAT* and *PCS* genes using the LightCycler^®^ 480 II thermocycler system (Roche, Switzerland). The primers for *Cu/Zn-SOD* (Yang et al., 2015), *POD* (L36157.1) (Helaoui et al., 2020), *CAT* (GU984379) (Desoky et al., 2019), *PCS* (AM407892.1) (Rahman et al., 2020a), and actin (JQ028730.1) (Rahman et al., 2020b) were found in **Table S1**. The actin gene was used as an internal reference to standardize the expression level of four genes. The details in PCR amplification procedures were shown in **Table S2**. Additionally, a heat melt procedure was performed to examine PCR specificity. Three technical replicates and three biological replicates were performed for all samples. The 2^-ΔΔCT^ method was used to calculate the relative quantity of transcripts (Livak and Schmittgen, 2001).

### Analysis of Cys, GSH, PCs, soluble proteins, MDA and AsA contents, and SOD, POD, CAT, and APX activities in plants

The sample solution for the determination of Cys, GSH, PCs, and MDA was prepared as described by Jia et al. (2018). Contents of Cys and GSH were examined according to Jia et al. (2018). The content of PCs was determined by the method provided by Chen et al. (2014). Malondialdehyde content was examined using thiobarbituric acid with spectrophotometer (Guo et al., 2007). Soluble proteins were extracted according to Yang et al. (2020), and the content was determined according to Bradford (1976). Fresh tissues (0.3 g) were homogenized using 1.5 mL trichloroacetic acid (5%, w/v) and centrifuged at 4,000×g at 4°C for 10 min. The AsA in the supernatant was determined spectrophotometrically by using 4,7-biphenyl-1,10-phenanthroline (BP) as the reagent (Arakawa et al., 1981). The enzyme solution for the analysis of SOD, POD, CAT, and APX activities was extracted according to Wang et al. (2022b). Superoxide dismutase activity was determined using photoreduction method of nitroblue tetrazolium (NBT) (Guo et al., 2007). Guaiacol colorimetry described by Guo et al. (2007) was used to examine POD activity. The CAT activity was determined by monitoring the changes in H_2_O_2_ concentration at 240 nm with time (Guo et al., 2007), and APX activity was examined by monitoring the decrease in AsA absorbance at 290 nm with time (Zhang et al., 2010).

### Data analysis

Two-way analysis of variance (ANOVA) was used to test the effects of temperature and Cd on all plant parameters. One-way ANOVA was used to examine the effect of *G. mosseae* on all plant parameters under ET + Cd. Duncan’s test was proceeded to test the differences between different treatments at 0.05 level when the ANOVA result was significant. Pearson correlation analysis was performed to evaluate the correlation between defense system in the shoots and that in the roots. All statistical analyses were conducted by SPSS 25.0 (SPSS Inc.). Redundancy analysis (RDA) were proceeded to assess the explanation of growth parameters to alfalfa defenses using Canoco 5.0.

## Results

### Plant biomass and photosynthetic pigments

**Figure 1:**
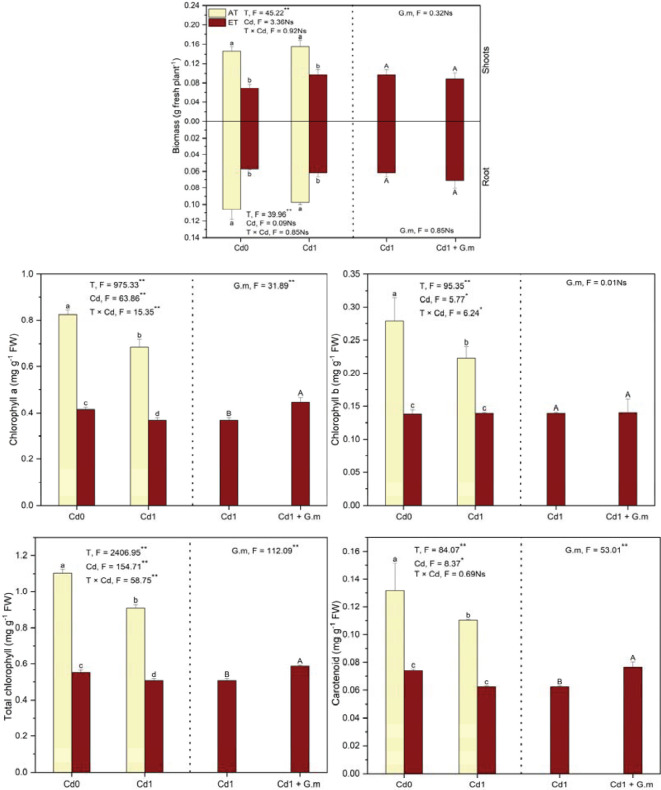
The biomass and photosynthetic pigments in alfalfa under different treatments tested by variance analysis (ANOVA) and summary of the results of ANOVA (F values and significance levels). G.m represented *Glomus mosseae*. Cd0 and Cd1 represented the control (no added Cd) and Cd pollution (added 8.0 mg Cd kg^-1^ dry weight soil), respectively. ET and AT represented elevated and ambient temperature, respectively. The T represented temperature. Different lowercase letters represented significant differences (*p* < 0.05) between the control, Cd, ET and ET + Cd. Different capital letters represented significant differences (*p* < 0.05) between ET + Cd + G.m and ET + Cd. Bars showed standard error (n = 3). ** and * represented significance at *p* < 0.01 and *p* < 0.05, respectively. The Ns represented not significant. The same below.

Relative to the control, ET (+ 3°C) decreased (*p* < 0.05) shoots (52.7%) and root biomass (45.8%) and contents of total chlorophyll (49.8%), chlorophyll a (49.7%) and b (50.3%), and carotenoids (43.7%), and Cd led to decreases (*p* < 0.05) in photosynthetic pigments (**Figure 1**). Elevated temperature decreased (*p* < 0.05) the biomass of the shoots (37.3%) and roots (37.0%), total chlorophyll (44.2%), and chlorophyll a (46.4%) and b (37.3%) contents under Cd exposure, however, *G. mosseae* colonization showed significant improvement on total chlorophyll, chlorophyll a, and carotenoid contents by 15.6%, 21.3%, and 23.2%, respectively, under ET + Cd (**Figure 1**). The effect of temperature on biomass was significant. Temperature, Cd, and their interaction showed significant effects on photosynthetic pigments, and *G. mosseae* significantly affected chlorophyll content under ET + Cd (**Figure 1**).

### Cadmium, N, S, P, and C contents in plants (shoots and roots)

**Figure 2:**
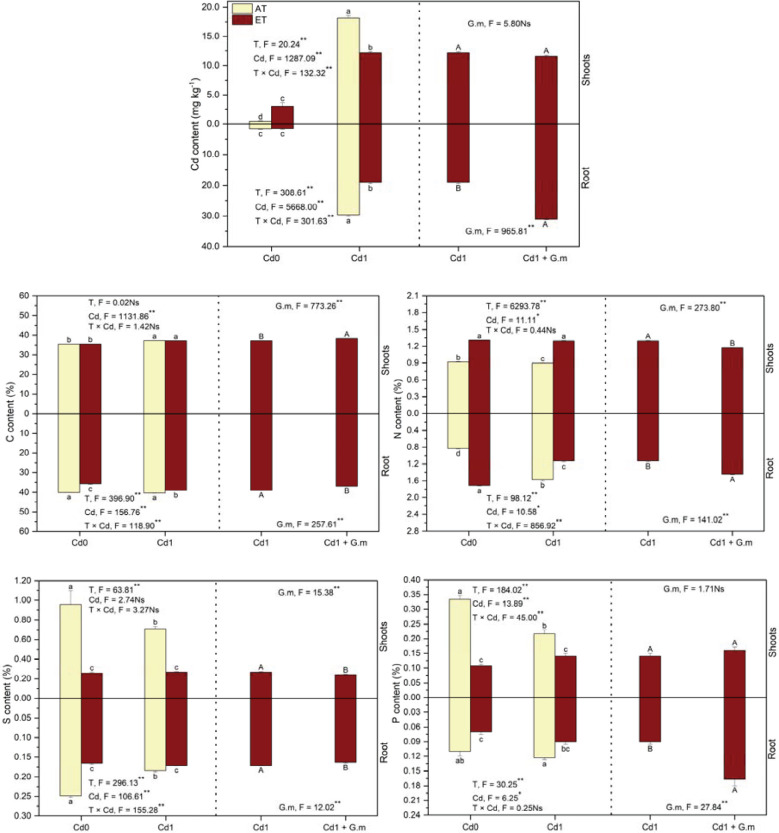
Cadmium, C, N, S, and P contents in alfalfa under different treatments tested by variance analysis (ANOVA) and summary of the results of ANOVA (F values and significance levels). Cd0 and Cd1 represented the control (no added Cd) and Cd pollution (added 8.0 mg Cd kg-1 dry weight soil), respectively. ET and AT represented elevated and ambient temperature, respectively. The T represented temperature. Different lowercase letters represented significant differences (p < 0.05) between the control, Cd, ET and ET + Cd. Different capital letters represented significant differences (p < 0.05) between ET + Cd + G.m and ET + Cd. Bars showed standard error (n = 3). ** and * represented significance at p < 0.01 and p < 0.05, respectively. The Ns represented not significant.

Elevated temperature decreased (*p* < 0.05) Cd uptake by plants by 34.4%, and *G. mosseae* colonization led to a significant increase in root Cd by 63.3% under Cd + ET (**Figure 2**). Compared to the control, C in the shoots and N increased (*p* < 0.05) by 5.0% and 88.0%, respectively, and S in the roots and N, S, and P in the shoots decreased (*p* < 0.05) by 25.8%, 2.2%, 25.8%, and 35.0%, respectively, under Cd exposure, and C (10.9%) in the roots and S (53.3%) and P (52.2%) in plants reduced (*p* < 0.05), and N in plants increased (*p* < 0.05) by 74.5% under ET (**Figure 2**). Elevated temperature led to (*p* < 0.05) enhancement in N by 44.6% in the shoots and decreases in S and P in plants by 34.7% and 31.2%, respectively, under Cd exposure, in addition, C and N in the roots decreased (*p* < 0.05) under ET + Cd relative to Cd exposure (**Figure 2**). Temperature significantly influenced N, S, P, and Cd in plants, and the interaction of temperature and Cd showed significant effects on P in the shoots and C, N, and S in the roots (**Figure 2**). *G. mosseae* colonization obviously enhanced C in the shoots by 3.0% and N and P in the roots by 28.9% and 85.2%, respectively, and decreased N and S in the shoots by 9.5% and 10.3%, respectively, and C and S in the roots by 5.5% and 4.9%, respectively, under ET + Cd (**Figure 2**). Furthermore, *G. mosseae* showed significant effects on C, N, and S in the shoots and all parameters in the roots exposed to ET + Cd (**Figure 2**). Overall, C content was lower in the shoots than in the roots, but S and P contents were greater.

### SOD, POD, CAT, and APX activities and relative expression of *Cu/Zn-SOD, POD*, and *CAT* genes

**Figure 3:**
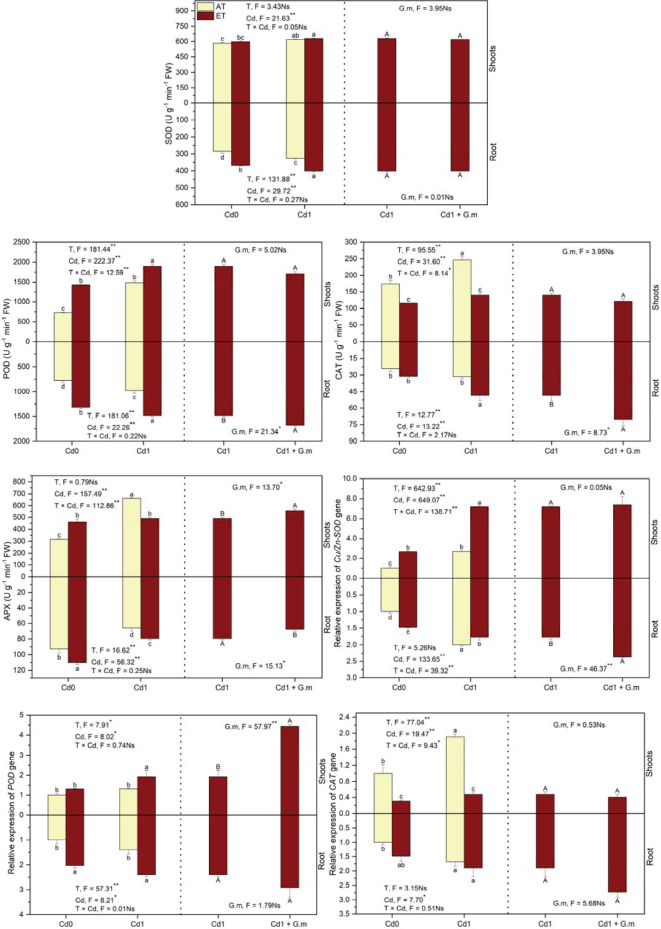
Activities and gene expression of antioxidant enzymes in alfalfa under different treatments tested by variance analysis (ANOVA) and summary of the results of ANOVA (F values and significance levels). SOD, POD, CAT, and APX represented superoxide enzyme, peroxidase, catalase, and ascorbate peroxidase, respectively. Cd0 and Cd1 represented the control (no added Cd) and Cd pollution (added 8.0 mg Cd kg-1 dry weight soil), respectively. ET and AT represented elevated and ambient temperature, respectively. The T represented temperature. Different lowercase letters represented significant differences (p < 0.05) between the control, Cd, ET and ET + Cd. Different capital letters represented significant differences (p < 0.05) between ET + Cd + G.m and ET + Cd. Bars showed standard error (n = 3). ** and * represented significance at p < 0.01 and p < 0.05, respectively. The Ns represented not significant.

Compared to the control, Cd and ET improved (*p* < 0.05) SOD, POD, and APX activities in plants by 10.2%, 64.8%, and 82.7%, CAT activity in the shoots increased (*p* < 0.05) by 40.9% under Cd exposure but decreased (*p* < 0.05) by 33.4% under ET alone, and activities of SOD (24.4%) and POD (125.3%) in plants, APX (55.0%) in the shoots and CAT in the roots increased (*p* < 0.05) by 99.8%, but APX activity in the roots decreased significantly by 14.2% under ET + Cd (**Figure 3**). Additionally, ET stimulated (*p* < 0.05) POD activity in plants by 39.6% and SOD, CAT, and APX in the roots by 23.0%, 53.8%, and 20.8%, respectively, under Cd exposure, however, CAT and APX activities in the shoots decreased (*p* < 0.05) by 43.3% and 26.0%, respectively, under ET + Cd relative to Cd alone (**Figure 3**). *G. mosseae* colonization led to a significant stimulation on activities of APX (13.4%) in the shoots and POD (13.0%) and CAT (46.5%) in the roots but reduced (*p* < 0.05) APX activity in the roots by 15.0% under ET + Cd (**Figure 3**). Temperature showed significant impact on activities of SOD, POD, and CAT in the shoots and SOD, POD, CAT, and APX in the roots, and significant effect of Cd on four enzymes activities in plants was observed. Additionally, temperature and Cd showed significant interaction on POD, CAT, and APX in the shoots. *G. mosseae* significantly affected APX in shoots and POD, CAT, and APX in the roots under ET + Cd (**Figure 3**). Overall, enzymes activities were greater in the shoots than in the roots.

Relative to the control, Cd significantly up-regulated gene expression of *Cu/Zn-SOD* and *CAT* in plants by 133.1% and 79.6%, respectively, ET up-regulated (*p* < 0.05) gene expression of *Cu/Zn-SOD* in plants by 106.4% and *POD* in the roots by 102.4%, ET + Cd led to increases (*p* < 0.05) in gene expression of *Cu/Zn-SOD* and *POD* in plants by 347.5% and 116.2%, respectively, and *CAT* in the roots by 89.3%, and CAT gene expression in the shoots distinctly decreased under the treatments of ET and ET + Cd by 69.2% and 52.9%, respectively (**Figure 3**). Elevated temperature significantly up-regulated gene expression of *Cu/Zn-SOD* in plants and *POD* in the roots by 169.3% and 58.8%, respectively, and down-regulated CAT gene expression in the shoots by 75.3% under Cd exposure (**Figure 3**). *G. mosseae* colonization only showed a significant up-regulation on gene expression of *POD* in the shoots and *Cu/Zn-SOD* in the roots by 130.3% and 33.5%, respectively (**Figure 3**). Temperature and Cd significantly influenced three genes in the shoots, and the significant interaction of temperature and Cd on *Cu/Zn-SOD* and *CAT* genes in the shoots was observed (**Figure 3**). Additionally, *G. mosseae* significantly affected gene expression of *POD* in the shoots and *Cu/Zn-SOD* in the roots exposed to the coexistence of ET and Cd (**Figure 3**). Overall, expression of *Cu/Zn-SOD* gene was lower in the roots than in the shoots, however, *CAT* gene was greater.

### AsA, GSH, and MDA contents

**Figure 4:**
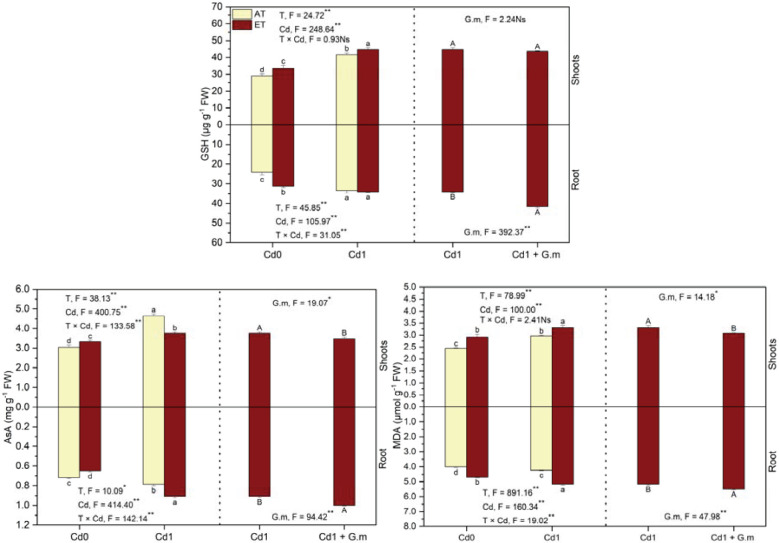
Contents of glutathione (GSH), ascorbic acid (AsA), and malondialdehyde (MDA) in alfalfa under different treatments tested by variance analysis (ANOVA) and summary of the results of ANOVA (F values and significance levels). Cd0 and Cd1 represented the control (no added Cd) and Cd pollution (added 8.0 mg Cd kg-1 dry weight soil), respectively. ET and AT represented elevated and ambient temperature, respectively. The T represented temperature. Different lowercase letters represented significant differences (p < 0.05) between the control, Cd, ET and ET + Cd. Different capital letters represented significant differences (p < 0.05) between ET + Cd + G.m and ET + Cd. Bars showed standard error (n = 3). ** and * represented significance at p < 0.01 and p < 0.05, respectively. The Ns represented not significant.

Relative to the control, contents of GSH, AsA, and MDA in the shoots and GSH and MDA in the roots increased (*p* < 0.05) under ET, Cd, and ET + Cd, additionally, AsA in the roots increased significantly under Cd and ET + Cd by 9.5% and 26.4%, respectively, and decreased distinctly by 9.8% under ET (**Figure 4**). Elevated temperature enhanced (*p* < 0.05) contents of GSH (7.2%) and MDA (11.1%) in the shoots and AsA (15.5%) and MDA (22.0%) in the roots and led to a significant decrease in AsA in the shoots by 19.4% under Cd exposure (**Figure 4**). *G. mosseae* colonization led to significant decreases in AsA and MDA in the shoots by 7.4% and 6.5%, respectively, and increases in GSH, AsA, and MDA in the roots by 22.2%, 10.3%, and 6.6%, respectively, under ET + Cd (**Figure 4**). The interaction of temperature and Cd had a significant effect on AsA in plants, MDA and GSH in the roots, and *G. mosseae* significantly affected AsA and MDA in the shoots and three compounds in the roots under ET + Cd (**Figure 4**). Additionally, GSH and AsA contents were greater in the shoots than in the roots, however, MDA content was lower.

### Cys and PCs contents and relative expression of *PCS* gene

**Figure 5:**
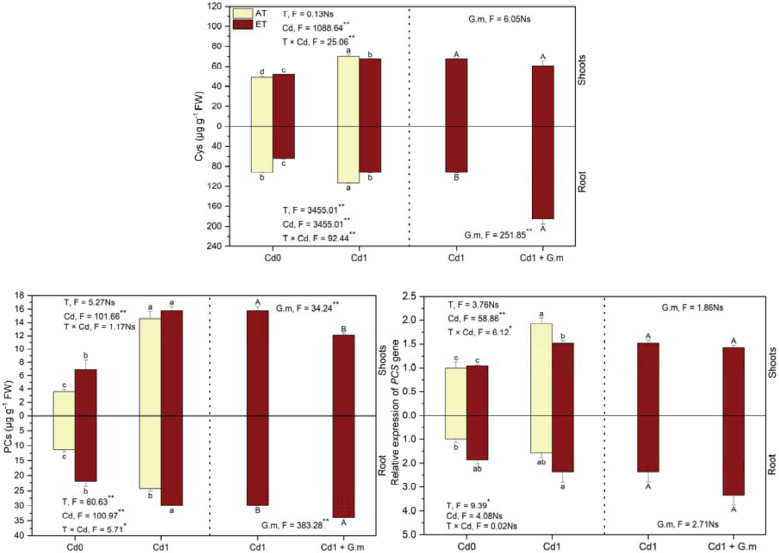
Cysteine (Cys) and phytochelatins (PCs) contents and relative expression of phytochelatins synthase genes (*PCS*) in alfalfa under different treatments tested by variance analysis (ANOVA) and summary of the results of ANOVA (F values and significance levels). Cd0 and Cd1 represented the control (no added Cd) and Cd pollution (added 8.0 mg Cd kg-1 dry weight soil), respectively. ET and AT represented elevated and ambient temperature, respectively. The T represented temperature. Different lowercase letters represented significant differences (p < 0.05) between the control, Cd, ET and ET + Cd. Different capital letters represented significant differences (p < 0.05) between ET + Cd + G.m and ET + Cd. Bars showed standard error (n = 3). ** and * represented significance at p < 0.01 and p < 0.05, respectively. The Ns represented not significant.

Contents of Cys in the shoots and PCs in plants increased significantly under Cd, ET, and ET + Cd relative to the control, by 42.1%, 5.1%, and 36.1%, respectively, and by 209.8%, 92.8%, and 251.1%, respectively, and ET led to a significant decrease in Cys in the plants by 11.1% and an increase in PCs in the roots by 23.1% under Cd exposure (**Figure 5**). Relative to the control, the relative expression of *PCS* gene in the shoots increased significantly under Cd and ET + Cd by 92.4% and 52.2%, respectively, and *PCS* gene expression in the roots obviously increased by 135.2% under ET + Cd (**Figure 5**). Elevated temperature down-regulated (*p* < 0.05) *PCS* gene expression in the shoots by 20.9% under Cd exposure (**Figure 5**). In addition, *G. mosseae* significantly decreased PCs content in the shoots by 23.2% and enhanced Cys and PCs contents in the roots by 101.0% and 13.8%, respectively, under ET + Cd (**Figure 5**). *G. mosseae* significantly influenced PCs in the shoots and PCs and Cys in the roots under the coexistence of ET and Cd (**Figure 5**). Overall, contents of two substances and *PCS* gene expression were lower in the shoots than in the roots.

### Soluble sugars and proteins contents

**Figure 6:**
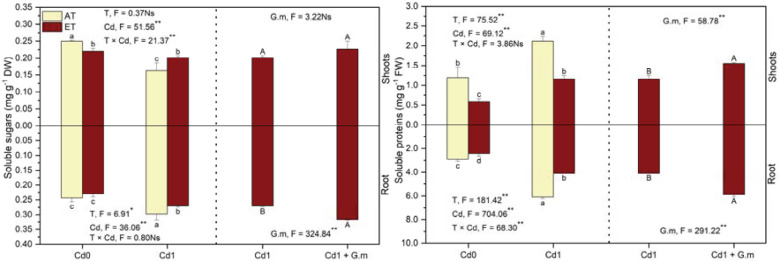
Soluble sugar and protein contents in alfalfa under different treatments tested by variance analysis (ANOVA) and summary of the results of ANOVA (F values and significance levels). Cd0 and Cd1 represented the control (no added Cd) and Cd pollution (added 8.0 mg Cd kg-1 dry weight soil), respectively. ET and AT represented elevated and ambient temperature, respectively. The T represented temperature. Different lowercase letters represented significant differences (p < 0.05) between the control, Cd, ET and ET + Cd. Different capital letters represented significant differences (p < 0.05) between ET + Cd + G.m and ET + Cd. Bars showed standard error (n = 3). ** and * represented significance at p < 0.01 and p < 0.05, respectively. The Ns represented not significant.

Compared to the control, soluble sugars in the shoots reduced significantly under all the treatments but soluble sugars in the roots significantly rose except for ET treatment, soluble proteins in the shoots increased significantly by 77.2% under Cd exposure and decreased significantly by 50.5% under ET, and soluble proteins in the roots rose (*p* < 0.05) under all the treatments except for ET (**Figure 6**). Elevated temperature enhanced (*p* < 0.05) soluble sugars in the shoots by 23.7% and decreased (*p* < 0.05) soluble proteins in plants and soluble sugars in the roots by 38.9% and 9.2%, respectively, under Cd exposure (**Figure 6**). *G. mosseae* enhanced (*p* < 0.05) soluble sugars in the roots and soluble proteins in plants by 17.5% and 38.6%, respectively, under ET + Cd (**Figure 6**). Overall, the contents of two substances were lower in the shoots than in the roots.

### Correlation of defense between shoots and roots

**Figure 7:**
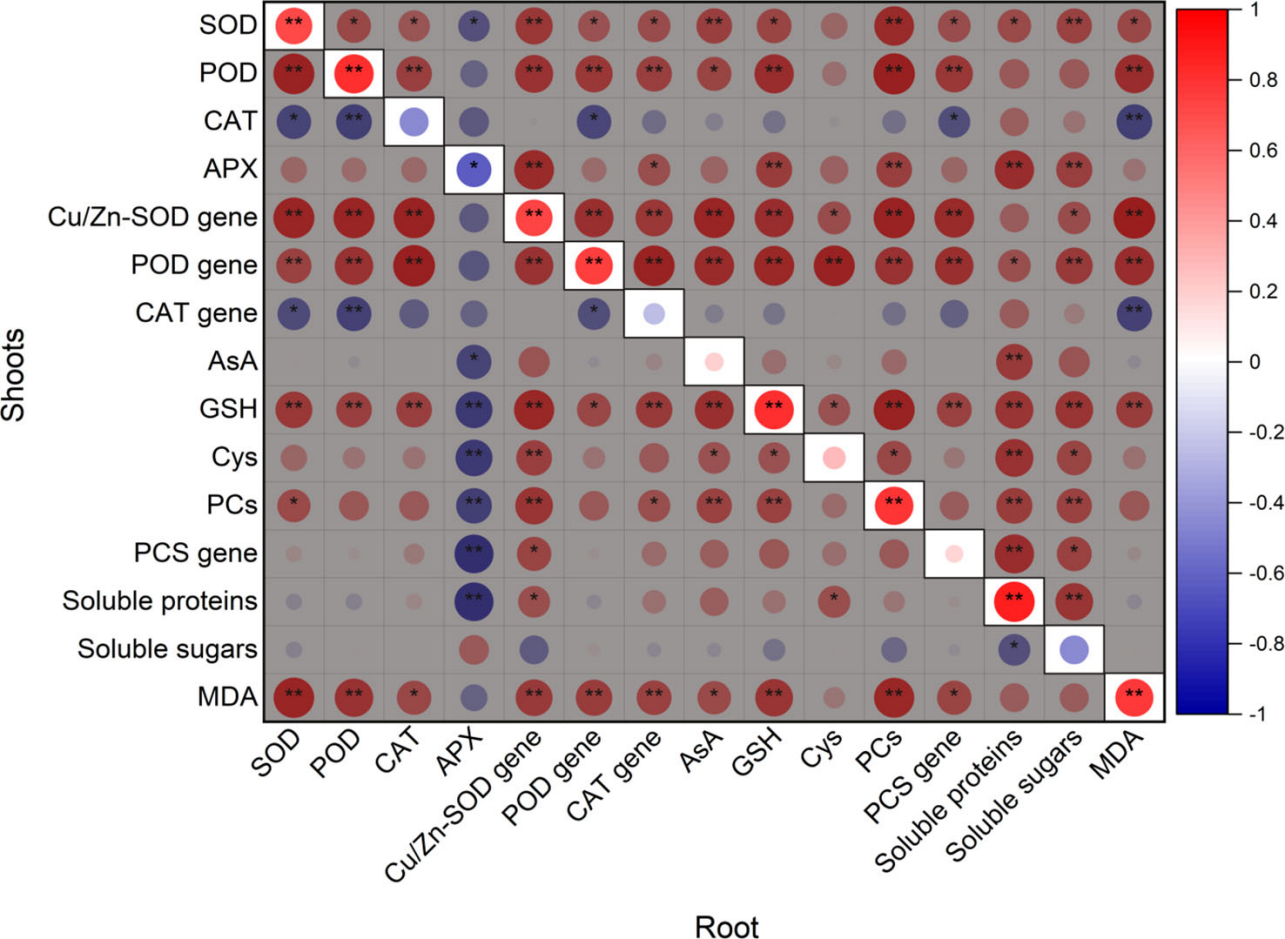
Pearson correlation between defense substances in root and those in shoots. ^*^ *p* < 0.05; ^**^ *p* < 0.05.

Except for POD activity in the shoots, enzymes activities and PCs content showed significant and positive correlation with the relative expression of their coding genes (**Table 1**). As showed in **Figure 7**, contents of GSH, PCs, soluble proteins, and MDA, activities of SOD and POD, and expression of *Cu/Zn-SOD* and *POD* genes in the shoots were significantly positively correlated with those in the roots, however, APX activity in the shoots was remarkably negatively correlated with that in the roots.

**Table 1:** Pearson correlation between PCs content and activities of superoxide enzyme (SOD), peroxidase (POD), and catalase (CAT) and expression of their encoding genes in alfalfa.

Items		Shoots				Root			
		*PCS*	*Cu/Zn-SOD*	*POD*	*CAT*	*PCS*	*Cu/Zn-SOD*	*POD*	*CAT*
Shoots	PCs content	0.788^**^							
	SOD activity		0.733^**^						
	POD activity			0.507					
	CAT activity				0.901^**^				
Root	PCs content					0.821^**^			
	SOD activity						0.675^**^		
	POD activity							0.909^**^	
	CAT activity								0.822^**^

^**^p < 0.01.

### Factors on alfalfa defense and the mechanism

**Figure 8:**
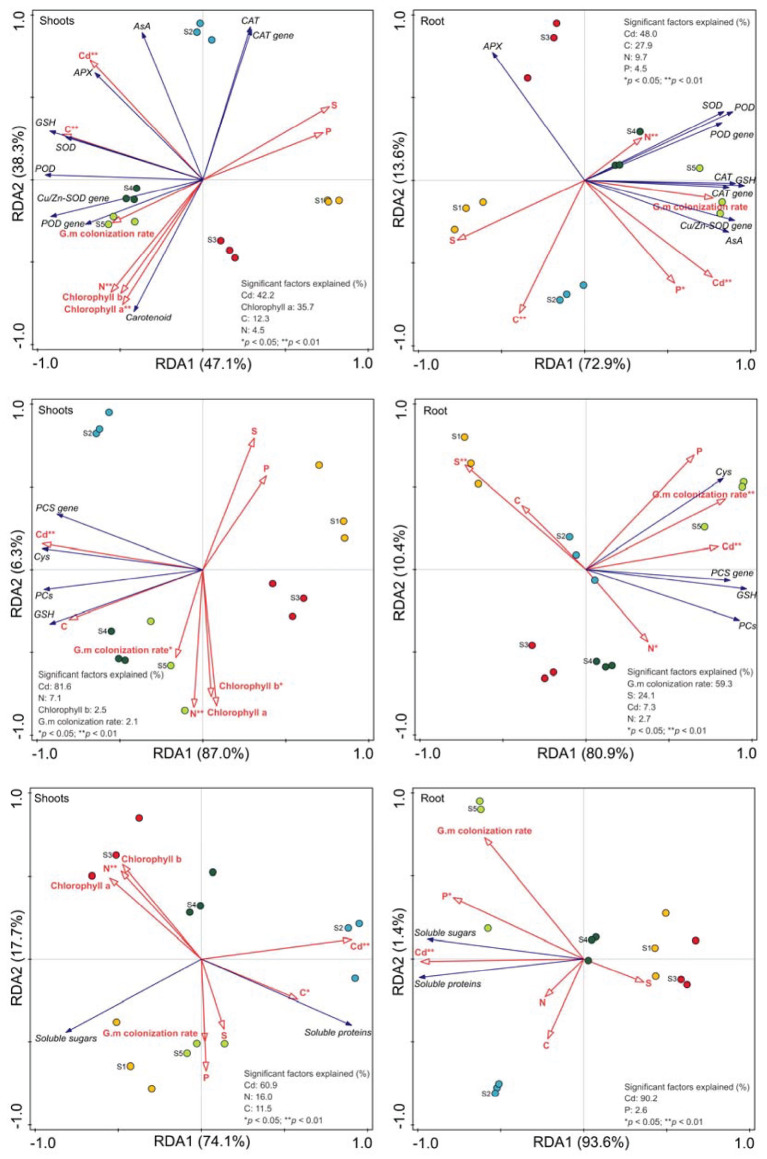
Redundancy analysis (RDA) on effects of Cd accumulation, *G. mosseae* colonization rate and alfalfa growth characteristics on defense system. G.m represented *Glomus mosseae*. S1-S5 represented the control, Cd, elevated temperature (ET), elevated temperature + Cd (ET + Cd) and elevated temperature + Cd + G.m (ET + Cd + G.m), respectively.

According to RDA data, plant growth characteristics, Cd uptake, and *G. mosseae* colonization rate explained 85.4% and 86.5% of the total variations of antioxidant system in the shoots and roots, respectively, 93.3% and 91.3% of the total variations of chelators in the shoots and roots, respectively, and 91.8% and 95.0% of the total variations of osmolytes in the shoots and roots, respectively (**Figure 8**). Cadmium, chlorophyll a, C, and N and Cd, C, N, and P were significant factors on antioxidant system in the shoots and roots, respectively, Cd, N, chlorophyll b, and *G. mosseae* colonization rate and *G. mosseae* colonization rate, S, Cd, and N significantly influenced chelators in the shoots and roots, respectively, and Cd, N, and C and Cd and P were significant factors on osmolytes in the shoots and roots, respectively (**Figure 8**). Additionally, the explanation ratio of Cd in plants was the greatest to the total variation for the shoots and the total variation of antioxidant system and osmolytes in the roots (**Figure 8**).

**Figure 9:**
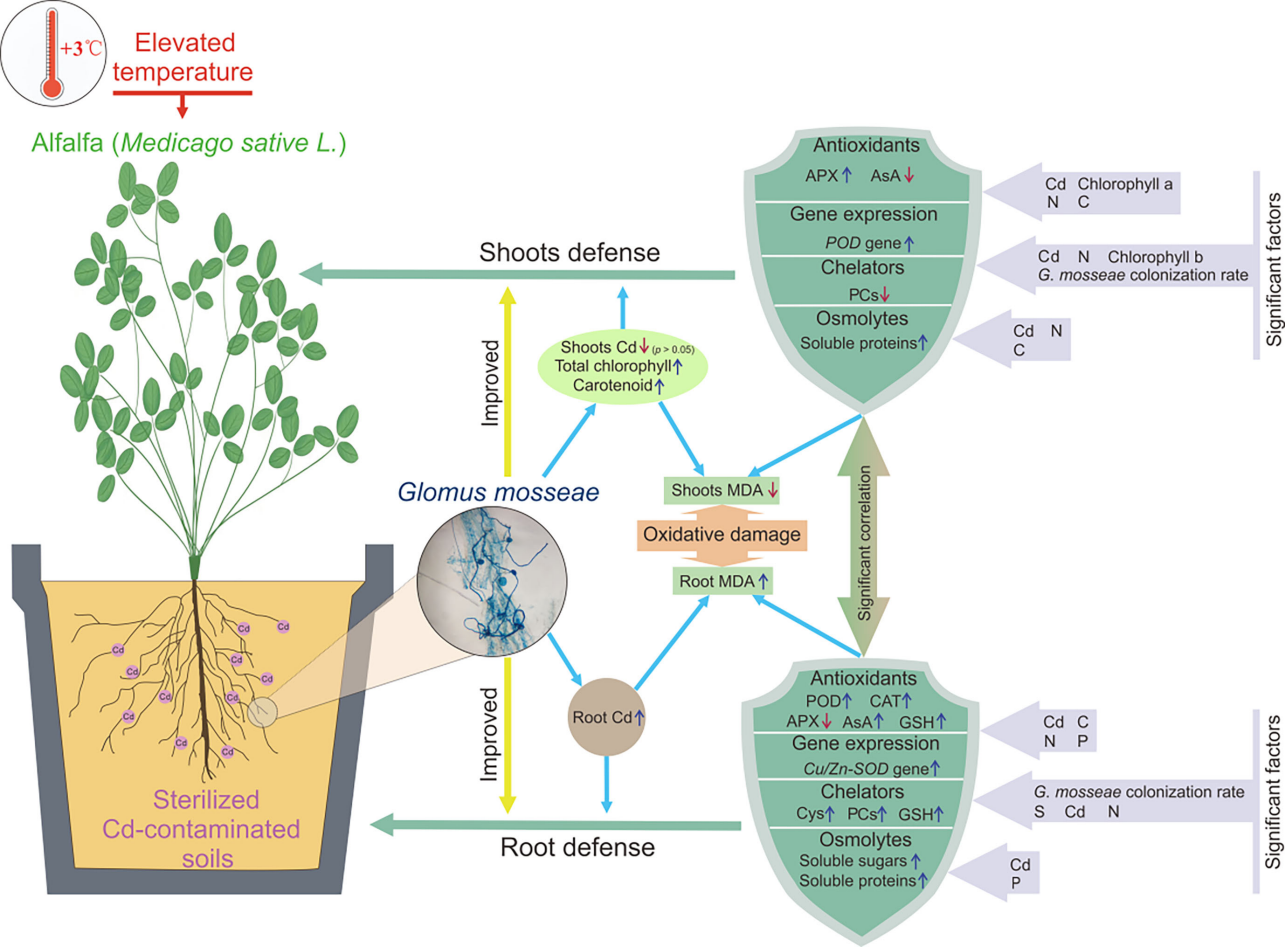
The mechanism on the improvement of *Glomus mosseae* on the adaptability of alfalfa to the coexistence of Cd-polluted soils and elevated air temperature.

According to the above analysis, *G. mosseae* generally improved the adaptability of alfalfa to the coexistence of Cd-polluted soils and elevated air temperature by enhancing the content of antioxidants, such as AsA, PCs, GSH, and Cys, inducing antioxidant enzyme gene expression, such as *Cu/Zn-SOD* and *POD*, stimulating the activities of antioxidant enzymes, such as APX, POD, and CAT, and enhancing the contents of chelators, such as PCs and GSH, and osmolytes, such as soluble proteins and sugars (**Figure 9**).

## Discussion

### Antioxidants in alfalfa

Cadmium can restrain or inactivate enzymes (such as SOD and POD) by binding the sulfhydryl group and replacing the metal cofactor (Fryzova et al., 2017). Additionally, Cd can induce the production of singlet oxygen and intracellular superoxide by interfering with the photosynthesis, and the reactive oxygen species (ROS) generated in this process can enhance antioxidant enzymes activities by regulating expression of encoding genes and stimulate generation of non-enzymatic antioxidants (Sharma and Dietz, 2009). Thus, lower chlorophyll and carotenoids under Cd exposure indicated that Cd interfered with the photosynthesis process, leading to greater activities of CAT and APX in the shoots, SOD, and POD and expression of *Cu/Zn-SOD*, *POD*, and *CAT* genes. However, significant up-regulation of *CAT* gene in the roots did not lead to distinct increase in CAT activity, which might be caused by CAT depletion generated by scavenging hydrogen peroxide and chelating Cd (Nozari et al., 2020). Additionally, CAT with insignificant changes, increased POD and decreased APX in the roots suggested that POD might be a main contributor for scavenging hydrogen peroxide. Higher AsA content might be another adaptive strategy to scavenge free radicals, which was consistent with the previous study (Dinakar et al., 2008). Overall, the antioxidant capacity of the shoots significantly influenced that of the roots according to Pearson correlation data (**Figure 7**) and was greater than that of the roots, in addition, increased MDA suggested that enzymatic and non-enzymatic antioxidants were not sufficient to scavenge ROS induced by Cd.

As described by previous studies (Wassie et al., 2020; Kareem et al., 2022), higher SOD, POD, and APX activities under ET relative to the control suggested that ET (+ 3°C) might cause oxidative damage to alfalfa, which led to decreased biomass, carotenoids, and P and increased MDA. High temperature can interfere with plant photosynthesis and affect enzyme activity in cells (Kaushal et al., 2016; Medina et al., 2021), which was consistent with our results. Additionally, carotenoids can effectively protect the chloroplast membrane structure and prevent membrane lipid peroxidation (Ahmad et al., 2010), which suggested that lower carotenoids under ET might cause the decreases in chlorophyll contents in this study. Thus, significant decreases in the biomass, chlorophyll, and carotenoids and increases in SOD, POD, and APX activities indicated that alfalfa suffered from ET, suggesting that a temperature rise of 3°C might cause reduction in plant production. The up-regulation of expression of *Cu/Zn-SOD* and *POD* genes under ET suggested that ET might activate the switches and control systems that regulate antioxidant enzymes genes, which contributed to the increases in enzymes activities. Lower CAT activity in the shoots was caused by the down-regulation of CAT gene expression, which might be associated with ROS-regulated signal transduction (Singh et al., 2019). Additionally, greater AsA and MDA also suggested that ET (+ 3°C) caused oxidative damage to alfalfa.

According to lower chlorophyll, carotenoids, biomass, C, S, and P under ET + Cd relative to Cd exposure, the coexistence of ET and Cd caused greater oxidation stress on alfalfa than Cd alone, which led to more MDA generation. Lots of MDA indicated that ET + Cd might result in higher accumulation of ROS in alfalfa, inducing higher expression of *Cu/Zn-SOD* in the shoots and *POD* genes compared to Cd alone, which was supported by the result from Wang et al. (2022a). Therefore, the up-regulation of gene expression of *Cu/Zn-SOD* in the shoots, *CAT* in the roots, and *POD* was the main factor on greater enzymes activities under ET + Cd relative to Cd. However, lower CAT and APX activities in the shoots under ET + Cd relative to Cd alone suggested that ET showed an inhibition on two enzymes probably by destroying the structural stability of proteins (Kaushal et al., 2016; Medina et al., 2021) or reducing substrate concentration.

Arbuscular mycorrhizal fungi can effectively protect the photosynthetic system of plants from heavy metals and heat by helping roots absorb nutrients and preventing the migration of heavy metals from soils to plants or from roots to leaves (Mathur et al., 2018; Yeasmin et al., 2019; Riaz et al., 2021). Here, higher N, P, and Cd in the roots indicated that *G. mosseae* improved the absorption of more nutrients by alfalfa and prevented Cd transferring from the roots to the shoots, which might reduce ROS generation in the shoots, leading to lower MDA and AsA and insignificant changes in SOD, POD, and CAT activities. Some studies have shown that AsA can increase rapidly under higher ROS accumulation (Akram et al., 2017; Wang et al., 2022a). Thus, higher total chlorophyll and carotenoids under G.m + ET + Cd relative to ET + Cd suggested that *G. mosseae* colonization might prevent ROS generation in alfalfa, which might be one reason for lower AsA in the shoots. However, *G. mosseae* colonization led to more Cd uptake by the roots, which improved the gene expression and activities of POD and CAT and contents of MDA and AsA under ET + Cd. Moreover, the trend of AsA was consistent with that of APX. The significant change in SOD activity did not happen although a significant up-regulation of *Cu/Zn-SOD* gene in the roots was observed, suggesting that Cd might bind the sulfhydryl group in SOD enzyme, which restrained or inactivated SOD activity (Fryzova et al., 2017). Additionally, *Fe-SOD* and *Mn-SOD* expression might down-regulate under *G. mosseae* colonization, being responsible for the insignificant change in SOD activity, which should be explored in the future. Overall, greater antioxidant enzymes activities and AsA content in the roots favored the defensive capacity, indicating that *G. mosseae* could improve the antioxidant ability of alfalfa, which agreed with the first hypothesis. According to higher chlorophyll and carotenoids, C in the shoots, and N in the roots under G.m + ET + Cd relative to ET + Cd, *G. mosseae* alleviated the oxidative stress caused by the coexistence of ET and Cd on photosynthesis, which was consistent with the second hypothesis.

### Chelators in alfalfa

Sulfur-containing chelators, such as PCs, GSH, and Cys, are ubiquitous detoxification agents described in wide variety of plants (Yadav, 2010; Capaldi et al., 2015; Cao et al., 2018), which are crucial for the survival of plants exposed to heavy metals. The PCs are synthesized non-translationally from GSH in a transpeptidation reaction catalyzed by *PCS*, which are a principal class of heavy metal chelators known in plants (Yadav, 2010; Gill and Tuteja, 2011). Thus, increased GSH and up-regulation expression of *PCS* gene under Cd exposure jointly led to greater PCs content in the shoots and roots. It has been found that Cd can activate the biosynthesis of Cys, GSH, and PCs (Heiss et al., 2003; Gill and Tuteja, 2011; Motaharpoor et al., 2019), which was consistent our results. As the basic constituent unit of GSH and PCs and possible rate-limiting factor on GSH biosynthesis (Gill and Tuteja, 2011), increased Cys was favorable for GSH and PCs synthesis, which would help alfalfa survival under Cd exposure. Overall, Cd significantly stimulated chelators synthesis, however, the production of chelators was not sufficient to capture all the Cd entering the cells, resulting in more MDA in plants.

Elevated temperature distinctly influences sulfur-containing chelators content by overproducing ROS which can induce GSH synthesis (Zagorchev et al., 2013; Hasanuzzaman et al., 2019). Here, increased MDA indicated that ET (+ 3°C) caused oxidative damage to alfalfa by producing lots of ROS, which induced the generation of more GSH, PCs, and Cys. Furthermore, the significant increase in PCs was caused by greater GSH and up-regulation of PCS under ET. However, lower S content indicated that alfalfa had suffered from ET.

Due to lower Cd uptake by plants under ET + Cd relative to Cd, increased GSH and PCs could be mainly attributed to ET (Kareem et al., 2022), of course, the effect of Cd could not be neglected (Heiss et al., 2003; Gill and Tuteja, 2011; Motaharpoor et al., 2019). Additionally, decreased Cys suggested that antioxidant enzymes might be key contributors for coping with the oxidative stress from ET and Cd.

Arbuscular mycorrhizal fungi can immobilize and sequester large amounts of Cd by hyphae, arbuscules, and vesicles (Janeeshma and Puthur, 2020), which could support higher Cd uptake by the roots under ET + Cd + G.m in this study. The stimulation of *G. mosseae* on Cd uptake by the roots activating the switch and control system of sulfur-containing chelating genes, which up-regulated *PCS* gene expression and effectively promoted the biosynthesis of GSH, Cys, and PCs under ET + Cd. Thus, *G. mosseae* colonization might improve alfalfa defense according to greater chelators in the roots, which was consistent with the first hypothesis. However, lower PCs in the shoots under ET + Cd + G.m relative to ET + Cd might be associated with the interception of Cd by *G. mosseae* and chelators in the roots, suggesting that *G. mosseae* could alleviate oxidative stress of Cd on the shoots. Overall, responses of GSH, Cys, and PCs and *PCS* gene expression to *G. mosseae* colonization were similar to that of Cd accumulation under Cd + ET, which suggested that the regulation of *G. mosseae* on chelators was achieved by regulating Cd uptake by alfalfa.

### Soluble sugars and proteins in alfalfa

Cadmium can inhibit or decrease plant photosynthesis by destroying the chloroplast membranes and reducing the content of photosynthetic pigments (He et al., 2008; Arshad et al., 2015), which can decrease sugar synthesis. Therefore, lower soluble sugars in the shoots might be caused by Cd accumulation. Lower soluble sugars and higher soluble proteins in the shoots suggested that soluble proteins might play major role in regulating osmotic capacity under Cd exposure. According to the RDA data, increased soluble sugars and proteins in the shoots were caused by Cd uptake, and higher contents in the roots than in the shoots might be associated with greater Cd accumulation. Cadmium might promote soluble proteins synthesis by stimulating the synthesis of various amino acids in plants, such as glutamic and alanine (Hsu and Kao, 2003), which could support increased soluble proteins in this study. Overall, the accumulation of soluble sugars and proteins might be against Cd-induced oxidative damage to alfalfa.

Decreased soluble sugars and proteins in alfalfa under ET relative to the control suggested that the oxidative stress of ET might be mainly scavenged by antioxidant enzymes and non-enzymatic antioxidants, which was supported by greater activities of SOD, POD, and APX and contents of GSH and PCs.

Under ET + Cd relative to Cd exposure, higher soluble sugars and lower soluble proteins in the shoots indicated that soluble sugars might play major role in regulating osmotic capacity, which was obviously different from that under Cd alone; however, soluble proteins might play key role for the roots. The results suggested that ET might affect or change species of substances regulating the osmotic in alfalfa, which might be associated with responses of Cd uptake, C, N, and P in plants to ET according to the RDA data.

Some studies have shown that AMF can improve contents of osmolytes, such as soluble sugars and proteins, in host plants exposed to Cd or heat stress (Wu, 2011; Dastogeer et al., 2022; Wang et al., 2022a), which was consistent with our results under ET + Cd. The stimulation of *G. mosseae* on two substances suggested that AMF might improve the osmotic regulation ability of alfalfa against oxidative stress from the coexistence of ET and Cd. Additionally, higher soluble sugars and proteins in the roots relative to shoots were caused by the significant stimulation of *G. mosseae* on Cd uptake by the roots according to the RDA data. Overall, *G. mosseae* colonization improved defense ability of alfalfa in view of osmolytes, which was consistent with the first hypothesis.

## Conclusion

The oxidative stress from the coexistence of Cd and ET on alfalfa was greater than that from Cd alone according to more MDA accumulation, which led to higher activities of SOD and POD and contents of GSH and PCs. However, *G. mosseae* colonization significantly enhanced Cd uptake by the roots and reduced Cd in the shoots under ET (+ 3°C), resulting in more MDA generation in the roots, which induced more production of AsA, GSH, Cys, and PCs and soluble sugars and proteins in the roots under ET + Cd. Overall, *G. mosseae* colonization obviously improved defense ability of alfalfa under ET + Cd by decreasing Cd uptake by the shoots and producing more non-enzymatic antioxidants including Cys, GSH, PCs, and AsA in the roots, osmolytes including soluble sugars in the roots and proteins, and antioxidant enzymes including POD and CAT in the roots and APX in the shoots. Furthermore, *G. mosseae* showed significant stimulation on alfalfa growth by enhancing chlorophyll content and N and P uptake by the roots under ET + Cd. The results could improve our understanding of regulation of AMF on the tolerance of plants to the coexistence of heavy metals and global warming and phytoremediation of heavy metal-polluted sites under global warming scenarios.

## Data availability statement

The original contributions presented in the study are included in the article/**Supplementary Material**. Further inquiries can be directed to the corresponding author.

## Author contributions

The raw data supporting the conclusions of this article will be made available by the authors, without undue reservation. YF-G was involved with writing, experiment and data analysis. XJ was involved with idea, experiment design, writing - reviewing and editing. YH-Z was involved with experiment design. X-YD, C-YZ and X-JF were involved with experiment. All authors contributed to the article and approved the submitted version.
