# Supplementary material for: Glomus mosseae improved the adaptability of alfalfa (Medicago sativa L.) to the coexistence of cadmium-polluted soils and elevated air temperature
Source: Front Plant Sci. 2023 Mar 9;14:1064732. doi: 10.3389/fpls.2023.1064732 (PMC10033771; doi:10.3389/fpls.2023.1064732)
Supplement: Supplementary file 1 [file Table_1.docx]

**Table S1** Primers for gene expression of enzymes in alfalfa using reverse transcription quantitative PCR.

| Genes | Forward primer (5' to 3') | Reverse primer (5' to 3') |
| --- | --- | --- |
| *Cu/Zn-SOD* | ATGTCAACTGGAGCACATTTCAATC | TGGACAACAACAGCCCTTCCTATG |
| *POD* | CGACGATTACGAGTGAGCAA | AATGGCACTTTCCAATCAGG |
| *CAT* | GGCTGCTTGAAGTTGTTCTCCT | CTGCTAGTACCTCCTGATCCGTT |
| *PCS* | ACCCTTCCTCCACCTTCAAT | CCAGGGTCAATAGCAAGAGC |
| *Actin* | ACCGGTGTGATGGTTGGTAT | GCCACACGAAGCTCATTGTA |
