# Supplementary material for: Glomus mosseae improved the adaptability of alfalfa (Medicago sativa L.) to the coexistence of cadmium-polluted soils and elevated air temperature
Source: Front Plant Sci. 2023 Mar 9;14:1064732. doi: 10.3389/fpls.2023.1064732 (PMC10033771; doi:10.3389/fpls.2023.1064732)
Supplement: Supplementary file 2 [file Table_2.docx]

**Table S2** Procedures for reverse transcription quantitative PCR for gene expression of enzymes in alfalfa.

| Genes | Procedures for dsDNA synthesis |
| --- | --- |
| *Cu/Zn-SOD* | Pre-denaturation at 95 °C for 5 min, followed by 40 cycles of a 15 s denaturation at 95 °C, 20s annealing at 50 °C, and 60 s elongation at 72 °C. |
| *POD* | Pre-denaturation at 94 °C for 3 min, followed by 40 cycles of a 30 s denaturation at 94 °C, 30 s annealing at 50 ~ 45 °C, and 60 s elongation at 72 °C. |
| *CAT* | Pre-denaturation at 94 °C for 3 min, followed by 40 cycles of a 30 s denaturation at 94 °C,30 s annealing at 51°C, and 60 s elongation at 72 °C. |
| *PCS* | Pre-denaturation at 95 °C for 30 s, followed by 40 cycles of a 5 s denaturation at 95 °C, 30 s annealing at 60 °C, and 45 s elongation at 72 °C. |
| *Actin* | Pre-denaturation at 95 °C for 30 s, followed by 40 cycles at a 5 s denaturation at 95 °C, 30 s annealing at 60 °C, and 10 s elongation at 72 °C. |
